# Raynaud’s Phenomenon of the Nipple: Epidemiological, Clinical, Pathophysiological, and Therapeutic Characterization

**DOI:** 10.3390/ijerph21070849

**Published:** 2024-06-28

**Authors:** Thaís Gomes Moreira, Giovana Mamede Castro, Jucier Gonçalves Júnior

**Affiliations:** School of Medicine, Universidade Federal do Cariri (UFCA), Barbalha 63048-080, Ceará, Brazil

**Keywords:** breastfeeding, Raynaud phenomenon, treatment, systematic review

## Abstract

Raynaud’s phenomenon of the nipple is a possible cause of pain and breastfeeding cessation in lactating women. However, there are still few studies on the characterization of this manifestation. Thus, we aim to develop a systematic review of the literature carried out between January 1992 and January 2024 in PubMed, Scopus, Web of Science, Virtual Health Library (VHL), and Portal de Periódicos da CAPES. Of the 438 articles, 19 met the eligibility criteria. The findings were divided by heuristic questions into two groups: “Epidemiological, pathophysiological, and clinical characterization of Raynaud’s Phenomenon of the nipple” and “Treatment of Raynaud’s Phenomenon of the nipple”. Raynaud’s phenomenon of the nipple is commonly primary, being more prevalent in the postpartum period, in women with a mean age of 32 years. The main triggers appear to be stress and temperature change. Generally, it is associated with a change in color and pain during breastfeeding. A calcium channel blocker was the most used medication with or without non-pharmacological measures.

## 1. Introduction

Raynaud’s phenomenon (RP) was first described by Maurice Raynaud in 1862. It is a complex, chronic, and multifactorial clinical manifestation that occurs due to reversible vasospasm in fingers, toes, nose, lips, and nipples, and it may be primary or secondary [1]. Primary RP often occurs after the age of 30, is not associated with pathologic ANA patterns or nailfold capillaroscopy changes and has no family history. Secondary RP is usually associated with pathologic ANA patterns and nailfold capillaroscopy changes and occurs after 30 years of age without a family history. Secondary RP is associated with other diseases such as scleroderma, systemic lupus erythematosus, Sjögren’s syndrome, rheumatoid arthritis, cryoglobulinemia, polyarteritis nodosa, lymphoproliferative diseases, thyroid diseases, and others [1,2,3].

It is estimated that 5% of the world’s population suffers from RP, 80 to 90% of whom have the idiopathic form. Up to 20% of cases occur in women, and the prevalence in pregnant women is also high [4,5,6,7].

RP is caused by reversible vasospasm of the arterioles. This leads to biphasic or triphasic color changes in the affected organs. Classically, RP can manifest with color changes such as (i) white, (ii) blue, and (iii) red [1,2,3,4,5,6,7].

The first description of RP of the nipples dates back to 1970 by Gunther and was expanded by Coates in 1992 when he described a woman with painful color changes during breastfeeding. It can be associated with smoking, alcohol, coffee, and certain medications such ergot derivatives [1,2,4,5]. Thus, RP of the nipple is a possible cause of pain diagnosis in lactating women [8], and it can lead to breastfeeding cessation. The pain causes anxiety, difficulties with breastfeeding, and a deterioration in the quality of life in the postpartum phase [3,6,7,9,10]. However, despite the seriousness of the manifestation and its repercussions for the mother and the baby, studies are still scarce.

Therefore, the aim was to develop a systematic review of the literature based on the following key question—what practical contributions does the current scientific literature offer concerning the epidemiological, pathophysiological, clinical, and treatment aspects of RP in breastfeeding? Despite technological advances and publications since 1992, there might still be a lack of more effective theoretical contributions to the presentation of the epidemiology, pathophysiology, clinical picture, and diagnosis of RP in breastfeeding.

## 2. Material and Methods

### 2.1. Literature Review

A qualitative systematic review of the literature was conducted following the PRISMA protocol. Electronic databases, including PubMed, Scopus, Web of Science, Virtual Health Library (VHL), and Portal de Periódicos da Coordenação de Aperfeiçoamento Pessoal de Nível Superior (a virtual library created by the Brazilian Ministry of Health with restricted content to authorized users) were searched using the following strategy: #1. Raynaud Phenomenon (MeSH) AND breastfeeding (MeSH); #2. Raynaud Phenomenon (MeSH) AND breastfeeding (MeSH) AND treatment (MeSH); #3. Raynaud Phenomenon (MeSH) AND nipples (MeSH); #4. Raynaud disease (MeSH) AND breastfeeding (MeSH); #5. Raynaud disease (MeSH) AND breastfeeding (MeSH) AND treatment (MeSH); and #6. Raynaud disease (MeSH) AND nipples (MeSH), between January 1992 and January 2024. The year 1992 was chosen as the starting point because it marked the publication of the first paper describing the disease.

The study was carried out with the acronym PICOS, where “P” means patients with RP; “I” means epidemiological, pathophysiological, and clinical characterization of breast RP; “C” means healthy Control Group; and “O” means the outcomes of the clinical and epidemiological data.

### 2.2. Data Collection

Data were collected for the period from January 1992 to January 2024. The articles were pre-analyzed based on their titles and abstracts. Two researchers collected data individually, with a third senior researcher being responsible for evaluating discrepancies and doubts. After this selection, each article was read in full and its most relevant findings were placed in Table 1, with information about the authors, year of publication, country, patients (*n*), age (mean), study type, qualitative assessment, when RP appears, type of RP, type of color change, previous clinical symptoms, and treatment.

To analyze the quality of each study, for the convenience of the authors, the Study Quality Assessment Tool (https://www.nhlbi.nih.gov/health-topics/study-quality-assessment-tools, accessed on 3 June 2024), created by the National Heart, Lung, and Blood Institute (NHLB), was used. Case report studies were evaluated using the Quality Assessment Tool for Case Series Studies, and clinical trials were assessed using the Quality Assessment of Controlled Intervention Studies. These tools classify studies as “good”, “fair”, or “poor” based on the presence or absence of relevant methodological elements for each type of study.

### 2.3. Eligibility Criteria

For each search strategy, the following screening was performed in this order: (i) review articles or short commentaries, editorials, communications, and letters to the editor; (ii) papers with animal models or dissertations were excluded.

Secondly, the following articles were selected: (i) articles in English, Portuguese, and Spanish; (ii) articles that were online and fully available in databases; (iii) articles that dealt specifically with RP in the nipple.

Finally, those selected articles that were classified as “poor” when analyzed with the Study Quality Assessment Tool were excluded.

### 2.4. Ethics

Considering this is a systematic literature review, Resolution 510/16 of the Brazilian National Health Council (CNS, acronym in Portuguese) dismisses the need for approval by a human research ethics committee. This review was registered with the PROSPERO platform under the number CRD42024543379.

## 3. Results

**Figure 1:**
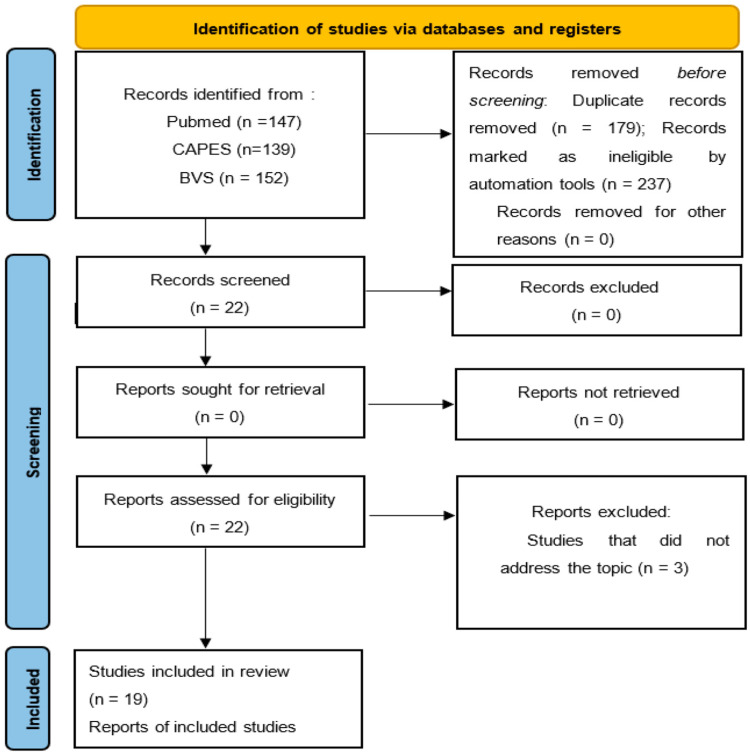
PRISMA flow diagram.

According to the research strategy (Figure 1), 438 articles were identified, of which 19 were selected. The findings were divided by heuristic questions into two groups: “Epidemiological, pathophysiological, and clinical characterization of RP of the nipple” and “Treatment of RP of the nipple”.

All reviewed studies were case reports or case series. Of these, at least 52.6% belonged to a sample from Europe (UK, England, Spain, Portugal, Germany, Denmark, and Norway), followed by North America (USA) with 42.1%, and Oceania (Australia) with 5.3% (Table 1).

In the quality analysis, more than half (63.1%) were classified as “good”, the others being “fair” (Table 1).

**Figure 2:**
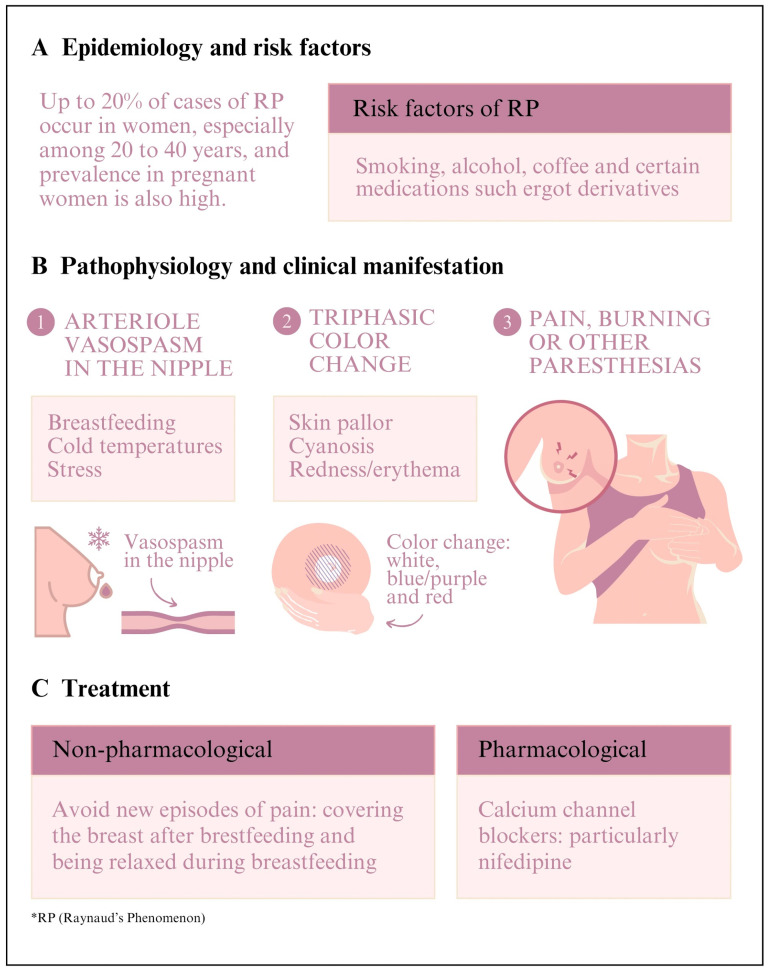
Raynaud’s phenomenon of the nipple: epidemiology, risk factors, pathophysiology, clinical manifestation, and treatment [1,2,3,4,5,6,7,8,9,10,11,12,13,14,15,16,17,18,19,20,21,22,23,24,25,26,27,28,29,30,31,32,33,34,35,36,37,38] (Adapted).

According to data from the literature, 29 patients with RP of the nipple were found. All of them were female, with a mean age of 32 years, ranging from 24 to 41 years. Most patients with this disease were postpartum (75.8%), and the remaining patients were pregnant (Figure 2).

The most common clinical signs were nipple pain associated with a change in color caused by vasospasm at low temperatures or stressful moments (Figure 2). Those changes in nipple color were triphasic (48.27%)—changing from white to blue/purple, and then, to red; biphasic (41.37%)—changing from white to blue/purple or red; and unspecified for the remaining of the cases (10.34%).

Most of the patients had primary RP (82.75%) of whom 58.33% demonstrated symptoms of the condition before pregnancy, such as vasospasm of the fingers, toes, and/or nipples with purplish color during cold weather or distressful moments.

Secondary RP was shown in 17.24% of the cases, with 75% (four cases) with symptoms before pregnancy and 25% (one case) without previous signs.

The findings in Table 1 and Figure 2 show that the most acceptable drug treatment for nipple pain caused by vasospasm was nifedipine (48.27%), which was frequently described in the studies. It was administered at doses of 30 mg twice a day, showing good recovery from the symptoms. Other treatments included non-pharmacological measures (20.68%), namely explaining the condition to the women and clarifying the cause of the symptoms, probably because the trigger for the RP symptoms in those cases was stress, and the remaining cases were unspecified (31.03%). Only two patients refused treatment with nifedipine, and the treatment was unspecified in seven other cases.

**Table 1:** Main findings.

Author (Year)-Country	Study Type- Quality Assessment	Patients (*n*)	Age (Years)	When RP Appears	Type of RP	Type of Color Change	Previous Clinical Symptoms	Treatment
Ferrando et al. (2023) [3] - Spain	Case report - Fair	1	39	1 week postpartum and 12 months postpartum	Secondary	Unspecified	Yes	Nifedipine
Morino and Winn (2007) [4] - USA	Case report - Fair	1	24	27 days postpartum	Primary	Triphasic	Yes	Non- Pharmacological
McGuinness and Cording (2013) [5] - England	Case report - Good	1	37	34 weeks of pregnancy	Secondary	Triphasic	Yes	Weaning Labetalol postpartum
Holmen and Backe (2009) [8] - Norway	Case report - Good	1	25	Beginning of second trimester of pregnancy	Primary	Triphasic	Yes	Nifedipine
Lawlor-Smith and Lawlor-Smith (1997) [9] - Australia	Case series - Fair	5	28	1 week postpartum	Primary	Triphasic	No	Unspecified
			30	3 weeks postpartum	Primary	Triphasic	Yes	Unspecified
			32	2 weeks postpartum	Primary	Biphasic	Yes	Unspecified
			32	1 week postpartum	Primary	Biphasic	No	Unspecified
			30	day postpartum	Primary	Biphasic	No	Unspecified
Gallego and Aleshaki (2020) [10] - USA	Case report - Good	1	36	3 weeks postpartum	Primary	Triphasic	No	Nifedipine and Pregabalin
Hardwick, McMurtrie and Melrose (2002) [15] - UK	Case report - Fair	1	41	16 weeks of pregnancy	Secondary	Biphasic	No	Non- Pharmacological
Stammler, Lawall and Diehm (2003) [16] - Germany	Case report - Good	1	38	13 weeks of pregnancy	Secondary	Triphasic	Yes	LMWH and AAS
Jansen and Sampene (2019) [17] - USA	Case series - Good	2	40	17 weeks of pregnancy	Secondary	Triphasic	Yes	Substitution of Labetalol for Nifedipine
			32	Postpartum	Primary	Triphasic	Yes	Non- Pharmacological
O’ Sullivan and Keith (2011) [19] - USA	Case report - Good	1	32	Immediately postpartum	Primary	Biphasic	Yes (previous diagnostic)	Nifedipine and Nitroglycerin ointment
Wu, Chason and Wong (2012) [20] - USA	Case series - Good	2	23	3 months postpartum	Primary	Biphasic	No	Nifedipine
			32	Postpartum	Primary	Biphasic	Yes	Nifedipine
Laursen and Rørbye (2013) [21] - Denmark	Case report - Fair	1	33	35 weeks of pregnancy	Primary	Triphasic	Yes	Calcium channel blocker
Di Como et al. (2020) [22] - USA	Case report - Fair	1	38	33 weeks of pregnancy	Primary	Triphasic	Yes	Non- Pharmacological
Page and McKenna (2006) [31] - USA	Case report - Good	1	26	5 weeks postpartum	Primary	Biphasic	No	Nifedipine
Javier et al. (2012) [32] - Spain	Case report - Fair	1	28	2 weeks postpartum	Primary	Biphasic	No	Nifedipine
Quental et al. (2023) [33] - Portugal	Case report - Good	1	29	2 months postpartum	Primary	Biphasic	No	Nifedipine
Anderson, Held and Wright (2004) [34] - USA	Case series - Good	3	28	3 weeks postpartum	Primary	Unspecified	No	Refused (Nifedipine)
			31	2 weeks postpartum	Primary	Biphasic	No	Non- Pharmacological
			35	Immediately postpartum	Primary	Biphasic	No	Nifedipine
Abrantes et al. (2016) [38] - Portugal	Case series - Good	3	39	2 weeks postpartum	Primary	Triphasic	No	Nifedipine
			30	2 weeks postpartum	Primary	Triphasic	No	Refused
			31	6 weeks postpartum	Primary	Triphasic	No	Non- Pharmacological

AAS: aspirin; LMWH: low-molecular-weight-Heparin; RP: Raynaud’s phenomenon; UK: United Kingdom; USA: United States of America.

## 4. Discussion

### 4.1. Epidemiological, Pathophysiological, and Clinical Characterization of Raynaud’s Phenomenon of the Nipple

RP of the nipple appears to be more common in females, in younger individuals, and those with a family history [7,11,12]. Some data have shown that the average age of onset in patients with primary RP is 14 years old, and only 27% of cases started around 40 years old. In contrast, cases of secondary RP have been shown to begin in adulthood [7,13,14].

These data seem to support the idea that primary RP is more likely to affect young people of reproductive age and secondary RP is more likely to affect older people [11]. In the cases found in the analysis, the age of the five patients with secondary RP ranged from 37 to 41 years [3,5,15,16,17], while the age of the other patients ranged from 24 to 39 years, as described in Table 1.

In 14 of the cases analyzed, patients had had similar symptoms previously [3,4,5,8,9,16,17,18,19,20,21,22], some during adolescence and others during their first pregnancies. However, only one of these patients had been diagnosed with RP in the toes and fingers before pregnancy [19]. Only one patient also reported a family history [18]. However, this is in contrast with the data found in the literature, in which family history is present in more than one-third of cases, with a fivefold increase in the risk of developing the condition if there is family history [23,24,25]. According to the analyzed cases, only 3.4% reported family history of RP, a fact that probably demonstrates the lack of investigation or even diagnosis of RP in the background and may indicate one of the deficiencies of the studied reports [18].

Arteriole vasospasm in RP is clinically manifested by skin pallor followed by cyanosis of the affected area and subsequent redness/erythema, thus characterizing a change in skin color [26,27,28]. The triphasic change is usually associated with pain, burning, or other paresthesia [29,30]. According to the analyzed cases, in pregnant and postpartum patients, the symptoms described by them were severe pain and changes in the color of the nipples, both worsened by breastfeeding and cold temperatures [8,10,15,16,20,21,22,31,32,33]. They can occur before, during, or after breastfeeding, probably due to contact with cold air and the baby’s mouth.

This clinical picture of breast pain can be confused in many cases with candidiasis mastitis, due to the burning pain and whitish coloration of the nipples that can be also caused by a fungal infection. Many women are treated with systemic antifungals to control candidiasis in the breast and also have their children treated with oral antifungals [34]. Nevertheless, studies have found the presence of *Candida albicans* in women with various risk factors for RP of the nipples, such as the use of oral contraceptives, steroids, previous antibiotics use, and the presence of vaginal candidiasis [38]. Inappropriate treatment with fluconazole—an antifungal drug commonly used to treat candidiasis—can exacerbate the condition of nipple vasospasm [18]. This is one of the causes of underdiagnosis of RP in the nipple [4,28], because the condition is usually not recognized by many physicians and is not specifically treated in the primary stages of symptoms becoming evident, as demonstrated in some of the case reports reviewed [4,31,32,33].

As a consequence of RP in the nipple, we can mention: (i) the presence of ulcers and cracks in the nipple; (ii) difficulty in breastfeeding, which can lead to breast engorgement, aggravation of pain and infections such as cellulitis; (iii) aggravation of maternal anxiety leading to cessation of breastfeeding [9,28,34], which is highly important for quality nutrition and efficient immune protection, in addition to the establishment of a relevant bond between the mother and child [34,35].

As the articles under review are all case reports/series, generalizations and extrapolations cannot be made.

### 4.2. Treatment of Raynaud’s Phenomenon of the Nipple

RP treatment is not uniform among physicians in the reviewed clinical cases, probably due to the multiple causes of nipple pain episodes in patients, which can be triggered by cold, stress, or nipple trauma [25]. For this reason, treatments are divided into non-pharmacological—by educating the patient about the disease and advising her on how to breastfeed to avoid new episodes of pain, such as breastfeeding in warm places, covering the breast after breastfeeding, and being relaxed during breastfeeding [4,15,17,22,34], and pharmacological—by using medications that relax the vessels and prevent vasospasm [3,8,10,16,17,18,19,20,21,31,32,33,34].

Hence, calcium channel blockers, such as amlodipine, felodipine, isradipine, nicardipine, nifedipine, nimodipine, and nisoldipine [36] seem to be the most common choices made by the doctors responsible for the cases, with nifedipine as the main representative, at doses ranging from 20 to 60 mg once a day [3,8,10,17,18,20,31,32,33,34,37]. Nifedipine works by reducing the intramembranous influx of calcium into the myocardial cells and smooth muscles of the coronary arteries and, with a more pronounced effect, in peripheral resistance vessels [37]. This mechanism allows vasodilation of the arteries and reduction of peripheral vascular muscle tone, preventing the vasospasm that causes RP. Consequently, caffeine, vasoconstrictors, alcohol, cocaine, estrogens, immunosuppressants, as well as nicotine, are not recommended during treatment [25]. Nevertheless, although the use of calcium channel blockers, particularly nifedipine, represents an efficacious alternative for vasospasms in the nipple, showing a reduction in the frequency of attacks and ischemia episodes [38], it is imperative to underscore that the administration of this medication can precipitate adverse effects, including hypotension, tachycardia, and dizziness [38].

According to the studied cases, pharmacological treatment with nifedipine seems to be sufficient to alleviate breast pain in most breastfeeding mothers. Only two of the reviewed cases [3,19] presented a patient with recurrence of breast pain after treatment with nifedipine. The first one treated with a dose of 20 mg once daily returned to suffer from nipple pain 12 months postpartum [3] and the second one was treated with nifedipine 30 mg, once daily, but her pain could only be relieved with nitroglycerin ointment 0.2% [19], showing that not only nifedipine, but also other similar drugs, could be good choices for physicians facing such cases.

Moreover, some reports [5,17] show an association between the use of Labetalol and the occurrence of Raynaud’s symptoms during pregnancy. Labetalol is an alpha and beta-adrenergic blocker that causes vasodilation, decreased vascular resistance, and decreased blood pressure and is widely used to control chronic hypertension. In one of the women [17], switching from Labetalol to nifedipine was sufficient to control hypertension and nipple pain, while in the other patient [5], stopping Labetalol at the end of pregnancy put an end to the vasospasms and stopped the patient’s pain. More studies are needed to prove the interaction of Labetalol with the conditions induced by Raynaud’s disease, but according to the presented cases, the interference of Labetalol in breastfeeding mothers was surely responsible for the reduction of symptoms associated with nipple vasospasm [5,17]. Other medications such as phosphodiesterase type 5 inhibitors are contraindicated during breastfeeding.

In addition, studies have shown the effect of supplementation with omega-3 and omega-6 fatty acids, pycnogenol, L-carnitine, and vitamin D on vasomotor tone. However, the studies are cross-sectional, the samples are small, and most are studies with secondary RP on the hands.

## 5. Limitations

As limitations of the present study, we can mention: (i) the small sample size of the studies; (ii) the absence of control groups; (iii) the lack of important information and homogenization in the descriptions of the reports; (iv) the sample formed by reports/series of cases, which leads to the inability to generalize; and (v) the absence of randomized controlled trials (RCTs), which prevents meta-analyses from being carried out. These limitations of the literature itself reinforce the need for the present review, while demonstrating the need for a more in-depth study of RP of the nipple.

## 6. Conclusions

RP of the nipple is an important cause of pain during breastfeeding, with primary RP being more common, especially among women aged 20 to 40 years old, during pregnancy and the postpartum period. Vasospasm can occur before, during or after breastfeeding, probably due to contact with cold air and the baby’s mouth. The clinical picture is severe pain in the breasts, which is accompanied by color changes and intensifies during breastfeeding and in cold temperatures. It is also an important differential diagnosis in candidiasis mastitis, which often leads to inadequate initial treatment and the persistence of symptoms in these women. Treatments include non-pharmacological approaches by educating the patient about the disease and advising her on how to breastfeed to avoid new episodes of pain, and pharmacological approaches by using drugs that relax the vessels and prevent vasospasm, such as calcium channel blockers or labetalol.

The literature reports raise the question of how many women give up breastfeeding due to nipple pain caused by undiagnosed vasospasm, which compromises the quality of feeding and effective immune protection for the newborn as well as mother–infant bonding. This emphasizes the importance of early diagnosis, clarification of the condition, and appropriate treatment of these patients for the benefit of the woman and the child.

Robust studies, with larger and longitudinal samples, are needed to investigate the natural history of the disease, its consequences, and the safety and efficacy of treatments, particularly during pregnancy and lactation.

## Data Availability

The original contributions presented in the study are included in the article, further inquiries can be directed to the corresponding author.

## References

[1] Devgire V., Hughes M. (2019). Raynaud’s Phenomenon. Br. J. Hosp. Med..

[2] Herrick A.L., Wigley F.M. (2020). Raynaud’s Phenomenon. Best Pract. Res. Clin. Rheumatol..

[3] Ferrando P.J., Ferrer J.G.M., Puig F.S., Garcia C.V. (2023). Una paciente con fenómeno de Raynaud del pezón e hipertiroidismo. Rev. Clin. Med. Fam..

[4] Morino C., Winn S.M. (2007). Raynaud’s Phenomenon of the Nipples: An Elusive Diagnosis. J. Hum. Lact..

[5] McGuinness N., Cording V. (2012). Raynaud’s Phenomenon of the Nipple Associated with Labetalol Use. J. Hum. Lact..

[6] Pauling J.D., Hughes M., Pope J.E. (2019). Raynaud’s Phenomenon-an update on diagnosis, classification and management. Clin. Rheumatol..

[7] Haque A., Hughes M. (2020). Raynaud’s Phenomenon. Clin. Med..

[8] Holmen O.L., Backe B. (2009). An underdiagnosed cause of nipple pain presented on a camera phone. BMJ.

[9] Lawlor-Smith L., Lawlor-Smith C. (1997). Vasospasm of the nipple—A manifestation of Raynaud’s Phenomenon: Case reports. BMJ.

[10] Gallego H., Aleshaki J.S. (2020). Raynaud Phenomenon of the Nipple Successfully Treated With Nifedipine and Gabapentin. Cutis.

[11] Kayser C., Corrêa M.J.U., Andrade L.E.C. (2009). Fenômeno de Raynaud. Rev. Bras. Reum..

[12] Garner R., Kumari R., Lanyon P., Doherty M., Zhang W. (2015). Prevalence, risk factors and associations of primary Raynaud’s Phenomenon: Systematic review and meta-analysis of observational studies. BMJ Open.

[13] Planchon B., Pistorius M.-A., Beurrier P., De Faucal P. (1994). Primary Raynaud’s Phenomenon. Age of onset and pathogenesis in a prospective study of 424 patients. Angiology.

[14] Orsini M., Catharino A.M.S., Silveira V.C., Reis C.H.M., De Sant M., Cardoso C.E. (2021). Raynaud’s Phenomenon after cold exposure: A case report. Int. J. Case Rep. Images.

[15] Hardwick J.C.R., McMutrie F., Melrose E.B. (2002). Raynaud’s syndrome of the nipple in pregnancy. Eur. J. Obstet. Gynecol. Reprod. Biol..

[16] Stammler F., Lawall H., Diehm C. (2003). Repeated attacks of pain in the nipple of a pregnant woman. Unusual manifestation of Raynaud’s Phenomenon. Dtsch. Med. Wochenschr..

[17] Jansen S., Sampene K. (2019). Raynaud Phenomenon of the Nipple: An Under-Recognized Condition. Obstet. Gynecol..

[18] Garrison C.P. (2002). Nipple vasospasms, Raynaud’s syndrome, and nifedipine. J. Hum. Lact..

[19] O’Sullivan S., Keith M.P. (2011). Raynaud Phenomenon of the nipple: A rare finding in rheumatology clinic. J. Clin. Rheumatol..

[20] Wu M., Chason R., Wong M. (2012). Raynaud’s Phenomenon of the nipple. Obstet. Gynecol..

[21] Laursen J.B., Rørbye C. (2015). Raynaud’s Phenomenon of the papilla mammae caused by breastfeeding. Ugeskr. Laeger..

[22] Di Como J., Tan S., Weaver M., Edmonson D., Gass J.S. (2020). Nipple pain: Raynaud’s beyond fingers and toes. Breast J..

[23] Wigley F.M., Flavahan N.A. (2016). Raynaud’s Phenomenon. N. Engl. J. Med..

[24] Bowling J.C., Dowd P.M. (2003). Raynaud’s disease. Lancet.

[25] Reilly A., Snyder B. (2005). Raynaud Phenomenon. Am. J. Nurs..

[26] LeRoy E.C., Medsger T.A. (1992). Raynaud’s Phenomenon: A proposal for classification. Clin. Exp. Rheumatol..

[27] Gayraud M. (2007). Raynaud’s Phenomenon. Jt. Bone Spine.

[28] Barrett M.E., Heller M.M., Stone H.F., Murase J.E. (2013). Raynaud Phenomenon of the nipple in breastfeeding mothers: An underdiagnosed cause of nipple pain. JAMA Dermatol..

[29] Cooke J.P., Marshall J.M. (2005). Mechanisms of Raynaud’s disease. Vasc. Med..

[30] Flavahan N.A. (2015). A vascular mechanistic approach to understanding Raynaud Phenomenon. Nat. Rev. Rheumatol..

[31] Page S.M., McKenna D.S. (2006). Vasospasm of the nipple presenting as painful lactation. Obstet. Gynecol..

[32] Muñoz F.J.M., Martos M.D.C. (2012). Fenómeno de Raynaud y el amamantamienteo doloroso. Rev. Clin. Med. Fam..

[33] Quental C., Brito D.B., Sobral J.M., Macedo A.M. (2023). Raynaud Phenomenon of the Nipple: A Clinical Case Report. J. Fam. Reprod. Health.

[34] Anderson J.E., Held N., Wright K. (2004). Raynaud’s Phenomenon of the nipple: A treatable cause of painful breastfeeding. Pediatrics.

[35] Neville M.C., Anderson S.M., McManaman J.L., Badger T.M., Bunik M., Contractor N., Crume T., Dabelea D., Donovan S.M., Forman N. (2012). Lactation and neonatal nutrition: Defining and refining the critical questions. J. Mammary Gland Biol. Neoplasia.

[36] Anderson P.O. (2020). Drug Treatment of Raynaud’s Phenomenon of the Nipple. Breastfeed. Med..

[37] Abrantes A., Djokovic D., Bastos C., Veca P. (2006). Fenómeno de Raynaud do mamilo em mulheres a amamentar: Relato de três casos clínicos. Rev. Port. Med. Geral Fam..

[38] Thompson A.E., Pope J.E. (2005). Calcium channel blockers for primary Raynaud’s Phenomenon: A meta-analysis. Rheumatology.

